# Serological evidence of arenavirus circulation among fruit bats in Trinidad

**DOI:** 10.1371/journal.pone.0185308

**Published:** 2017-09-27

**Authors:** Ashley Malmlov, Janine Seetahal, Christine Carrington, Vernie Ramkisson, Jerome Foster, Kerri L. Miazgowicz, Sandra Quackenbush, Joel Rovnak, Oscar Negrete, Vincent Munster, Tony Schountz

**Affiliations:** 1 Arthropod-borne and Infectious Disease Laboratory, Department of Microbiology, Immunology and Pathology, College of Veterinary Medicine and Biomedical Sciences, Colorado State University, Fort Collins, Colorado, United States of America; 2 Department of Microbiology, Immunology and Pathology, College of Veterinary Medicine and Biomedical Sciences, Colorado State University, Fort Collins, Colorado, United States of America; 3 Department of Preclinical Sciences, Faculty of Medical Science, The University of the West Indies, St. Augustine, Republic of Trinidad and Tobago; 4 Sandia National Laboratories, Biotechnology and Bioengineering, Livermore, California, United States of America; 5 Virus Ecology Unit, Laboratory of Virology, Rocky Mountain Laboratories, NIAID/NIH, Hamilton, Montana, United States of America; University of Texas Medical Branch at Galveston, UNITED STATES

## Abstract

Tacaribe virus (TCRV) was isolated in the 1950s from artibeus bats captured on the island of Trinidad. The initial characterization of TCRV suggested that artibeus bats were natural reservoir hosts. However, nearly 60 years later experimental infections of Jamaican fruit bats (*Artibeus jamaicensis*) resulted in fatal disease or clearance, suggesting artibeus bats may not be a reservoir host. To further evaluate the TCRV reservoir host status of artibeus bats, we captured bats of six species in Trinidad for evidence of infection. Bats of all four fruigivorous species captured had antibodies to TCRV nucleocapsid, whereas none of the insectivore or nectarivore species did. Many flat-faced fruit-eating bats (*A*. *planirostris*) and great fruit-eating bats (*A*. *literatus*) were seropositive by ELISA and western blot to TCRV nucleocapsid antigen, as were two of four Seba’s fruit bats (*Carollia perspicillata*) and two of three yellow-shouldered fruit bats (*Sturnira lilium*). Serum neutralization tests failed to detect neutralizing antibodies to TCRV from these bats. TCRV RNA was not detected in lung tissues or lung homogenates inoculated onto Vero cells. These data indicate that TCRV or a similar arenavirus continues to circulate among fruit bats of Trinidad but there was no evidence of persistent infection, suggesting artibeus bats are not reservoir hosts.

## Introduction

Tacaribe virus (TCRV) is a New World mammarenavirus first isolated in 1956 from a moribund great fruit-eating bat (*Artibeus literatus*) collected in Port-of-Spain, Republic of Trinidad and Tobago during a rabies virus surveillance program at the Trinidad Regional Virus Laboratory (TRVL) [1, 2]. Nineteen isolates were obtained from six great fruit-eating bats and five purported Jamaican fruit bats (*A*. *jamaicensis*) but only one of those isolates remains; TRVL-11573 isolated from the initial great fruit-eating bat. Serological investigation of more than 2,000 mammals found that only bats of the *Artibeus* genus had evidence of TCRV infection, suggesting that artibeus bats may be reservoir hosts. Work performed about 15 years later also identified seropositive artibeus bats, suggesting TCRV continued to circulate in Trinidad [3]. Recent phylogenetic studies suggest that *A*. *jamaicensis* bats in Trinidad and Tobago are likely a distinct species, the flat-faced fruit-eating bat (*A*. *planirostris*) [4]. TCRV is closely related to Junin and Machupo viruses [5], the etiologic agents of Argentine and Bolivian hemorrhagic fevers, respectively, and has caused at least one laboratory-acquired human infection [6], raising the possibility that TCRV has zoonotic potential.

**Fig 1:**
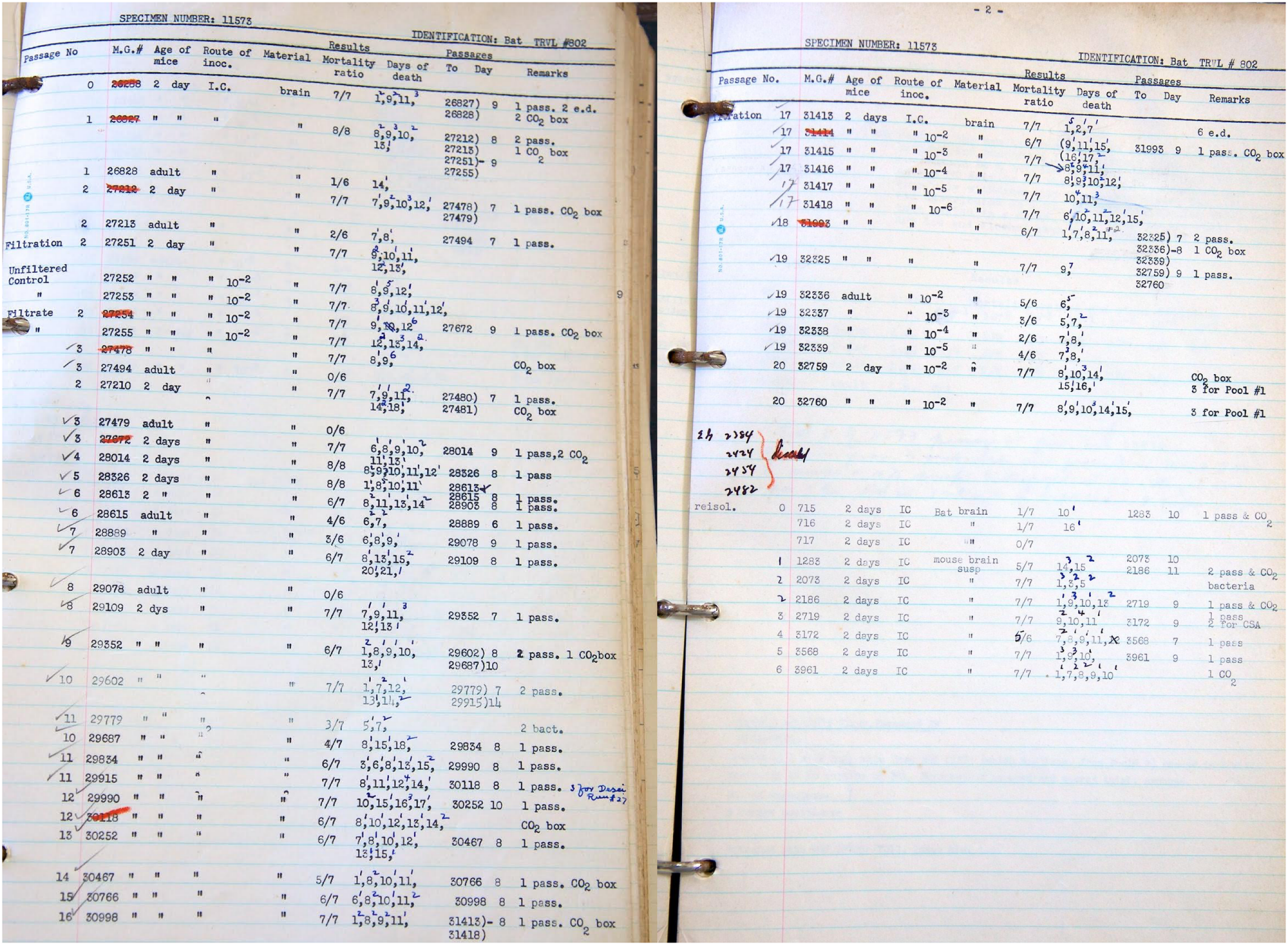
Passage history of Tacaribe virus isolate TRVL-11573 obtained from the library archive of the Caribbean Epidemiology Centre (CAREC), Port of Spain, Trinidad and Tobago. TCRV TRVL-11573 was isolated from bat TRVL #802 (great fruit-eating bat, *A*. *literatus*) and intracranially passaged in newborn mice (unknown strain). Twenty passages were made to generate the first stock of TCRV TRVL-11573.

Experimental infections of Jamaican fruit bats resulted in significant morbidity and mortality with neurological manifestations that included head tremors, inability to roost, remain upright or fly, and pathological involvement of lungs, liver, spleen and brain [7]. Several inoculated bats were asymptomatic, seroconverted with modest antibody titers by ELISA and low or no neutralization titers, and appeared to clear virus. These results suggested that infection leads to two outcomes; signs of disease that is fatal, or asymptomatic infection followed by clearance, neither of which is consistent with a typical arenavirus reservoir host. In 2014, TCRV was isolated from lone star ticks (*Amblyomma americanum*) collected in central Florida [8] where artibeus bats are not found. These isolates were genetically distinct from TRVL-11573 and include an additional 12 amino acids and several other polymorphisms in the glycoprotein [9]. However, it is likely that TRVL-11573 accumulated mutations during its 20 passages in suckling mice (Fig 1) and subsequent passage in Vero cells for preparation of viral stocks. Together, these data argue against artibeus bats as reservoirs of TCRV, and suggest that TCRV is likely hosted by another species, perhaps multiple species, and that spillover to bats results in disease.

We were interested in determining if artibeus bats are reservoir hosts of TCRV, to determine if the virus continues to circulate among bats in Trinidad and, if so, whether new isolates of the virus could be obtained. We collected blood and tissue samples from bats and performed serology, PCR and attempted virus isolation from tissues. Although many bats had antibodies reactive to recombinant TCRV nucleocapsid, none of the tested samples had neutralizing antibodies or viral RNA, and no isolates of TCRV were obtained. These findings are congruent with our experimental infection of Jamaican fruit bats and further indicates that bats may not be natural reservoir hosts of Tacaribe virus.

## Materials and methods

### Collection of bats

**Fig 2:**
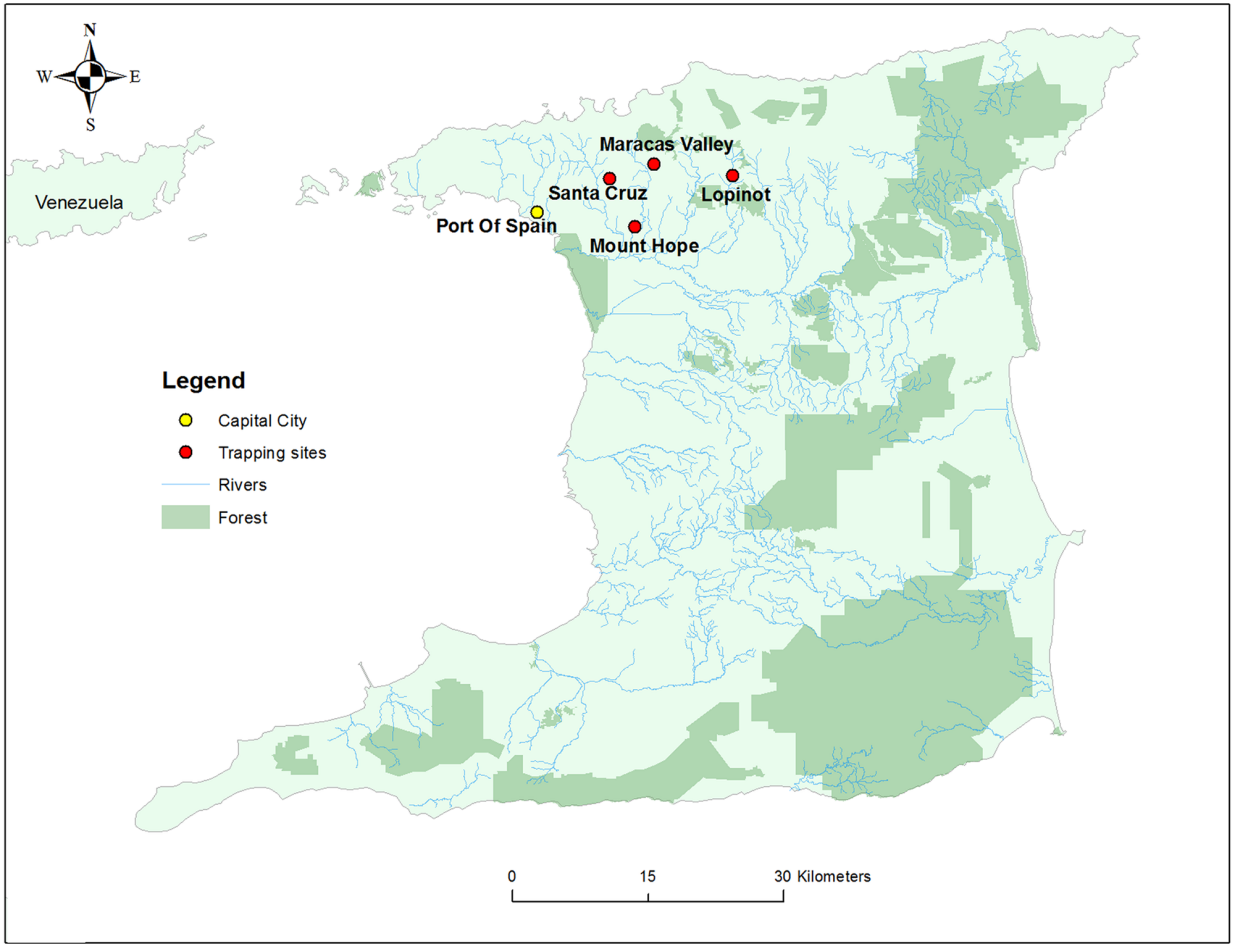
Locations of bat collections. Mt. Hope bats were collected during the day from a building roost on the campus of The University of West Indies. Collections from the remaining three sites were performed on three consecutive nights.

Bats were captured with mist nets in February, 2012 for sampling, with approval of the Ethics Committee, Faculty of Medical Sciences, University of the West Indies, St. Augustine Campus, and under a special game license from the Wildlife Section, Forestry Division, Ministry of Agriculture, Land and Fisheries, Republic of Trinidad and Tobago. Trap sites were Mount Hope (N 10.67120, W 061.28677), Lopinot (N 10.69792, W 061.32243), Santa Cruz (N 10.69596, W 061.44629), and Maracas Valley (N10.70945, W061.40177) (Fig 2). No threatened or endangered species were captured. Live bats were individually confined in cloth bags for transport to laboratory facilities at the University of the West Indies, St. Augustine for processing. Bats were humanely euthanized by inhalation of isoflurane and thoracotomy prior to tissue harvesting at necropsy. All personnel were immunized against rabies virus and appropriate PPE was worn during collections, and bat necropsies were performed in a class II biosafety cabinet. Samples were immediately frozen and stored at -80°C prior to shipment on dry ice to Colorado State University (Fort Collins, CO, USA) and Rocky Mountain Laboratories (Hamilton, MT, USA) for further processing. Species identification of artibeus bats was performed by direct sequencing of PCR products amplified with cytochrome b-specific primers (forward, ACCAATGACATGAAAAATCATCGTT; reverse, TCTCCATTTCTGGTTTTACAAGAC). Identification was determined by closest BLASTN matches in Genbank (nr database).

### Attempted virus isolation

Virus isolation was attempted from lung tissue, a target organ of TCRV-infected bats [7]. Tissues were individually placed into 1.5-ml screw cap tubes with 500 μl 5% FBS-DMEM and a 5 mm stainless steel ball, and homogenized on TissueLyser LT (Qiagen, Valencia, CA), for 5 min at 2 Hz to minimize heat and potential virus inactivation, placed on ice, homogenized again and placed on ice, and centrifuged at 3000 rpm for 5 min at 4°C. Four hundred microliters of the supernatant was added to 500 μl 5% FBS-DMEM and filtered through 0.2 μm Acrodisc filter and 100 μl of filtrate inoculated onto confluent Vero E6 cells in 24 well plates for 1 hour at 37°C, removed and 1 ml of 2% FBS-DMEM added. Medium was replaced on day 6. After 10 days of incubation, RNA was extracted from supernatants (QIAamp Viral RNA Mini kit) according to manufacturer’s instructions. Control TCRV (TRVL-11573) RNA was isolated from inoculated Vero E6 stocks.

### RNA extraction/PCR

The homogenized tissue pellets were used for RNA extractions by adding 350 μl RLT with 1% 2ME and homogenizing 2 x 5min at 50 Hz. RNA extraction was from the supernatant according to manufacturer’s instructions (RNeasy Mini kit with Qiashredders). All RNA samples were kept at -80°C for subsequent use.

RNA was reverse transcribed with random primers (Quantitect Reverse Transcription kit, Qiagen) according to manufacturer’s instructions. PCR was performed using TCRV primers (forward, 5’-TGTGGCTTTCTGAAGCAGTG-3’; reverse, 5’-AGGCTCTCGATCGCAAATTA) and PCR Core Kit (Qiagen) as previously described [7]. Amplification conditions were melting for 3 min at 94°C, 35 cycles at 94°C for 30 s, and annealing at 54°C for 30 s, and extension at 72°C for 1 min. Reactions were held at 72°C for 10 minutes. Samples were resolved on 1% agarose gels.

### Immunohistochemistry

Recombinant His-tagged TCRV nucleocapsid antigen [10] was expressed in *E*. *coli* BL21(DE3) cells and purified under denaturing conditions using TALON Metal Affinity Resin according to manufacturers’ protocol (Clontech Laboratories, Inc., Mountain View, CA). The TCRV NP was eluted in 300 mM imidazole, buffer exchanged into PBS and concentrated using a 30 kDa MW cut off Micron centrifugal filter. ELISA was performed as previously described using a protein-A/G-HRP conjugate [11]. Sera were diluted 1:100 in PBS for the ELISA. Samples with an absorbance of 3 times greater than the negative control serum absorbance were considered positive. Endpoint titers of positive samples were determined by ELISA, and antibody reactivity validated (1:500) by western blot. Ten micrograms of purified TCRV NP was separated on a 4–12% NuPage Bis-Tris one well polyacrylamide gel (Life Technologies/Invitrogen, Carlsbad, CA) and transferred to Immobilon-P membrane (Merck Millipore, Billeria, MA). The membrane was cut into 0.5 cm strips and incubated with sera diluted 1:100 in PBS overnight at 4°C. Membrane strips were washed, incubated with protein-A/G-HRP (diluted 1:5,000) and developed with 3,3’,5,5’-tetramethylbenzidine substrate (Kirkegaard and Perry Laboratories, Gaithersburg, MD). Control sera were from archived samples collected from a previous experimental infection of Jamaican fruit bats [7].

### Serum neutralization assay

TCRV TRVL-11573 was used for serum neutralization testing. Sera were diluted 1:10 in 2% FBS-DMEM in the first well and a log_2_ dilution series was prepared for each sample. TCRV (10^2^ TCID_50_) was added to each well in 100 μl (1:20 final dilution of serum) for 1 hour at 37°C, then the entire volume (200 μl) transferred to 96 well plates containing confluent Vero cells. Plates were scored for cytopathic effect on day 7.

## Results

Twenty-seven of the 62 adult bats were seropositive by initial ELISA screening and titers ranged from 100 to 800 (Table 1). However, western blot results indicated that only 22 of the bats had antibody specific to TCRV nucleocapsid antigen, and two artibeus bats (accession numbers 70 and 74) were ELISA-negative but WB-positive. Insufficient serum was available to perform WB analysis on bat 61 that was ELISA-positive. Antibodies were detected in some juveniles and may represent maternal antibody. Control sera from bats used in a previous experimental infection [7] had titers between 800 and 1600.

**Table 1:** Distribution and serology of bats in this study.

Animal ID	Site^1^	Species	Sex (M/F/U)^2^	Adult/ Juvenile	ELISA TT^3^	WB^4^
1	Mt. Hope	*A*. *planirostris trinitatis*	F	A	-	nd
2	Mt. Hope	*A*. *planirostris trinitatis*	U	J	-	nd
3	Mt. Hope	*A*. *planirostris trinitatis*	F	A	100	+
4	Mt. Hope	*A*. *planirostris trinitatis*	U	J	100	+
5	Mt. Hope	*A*. *planirostris trinitatis*	F	A	-	nd
6	Mt. Hope	*A*. *planirostris trinitatis*	F	A	-	nd
7	Mt. Hope	*A*. *planirostris trinitatis*	U	J	200	-
8	Mt. Hope	*A*. *planirostris trinitatis*	F	A	-	nd
9	Mt. Hope	*A*. *planirostris trinitatis*	F	A	200	+
10	Mt. Hope	*A*. *planirostris trinitatis*	F	A	100	+
11	Mt. Hope	*A*. *planirostris trinitatis*	F	A	-	nd
12	Mt. Hope	*A*. *planirostris trinitatis*	M	J	-	nd
13	Mt. Hope	*A*. *planirostris trinitatis*	M	J	-	nd
14	Mt. Hope	*A*. *planirostris trinitatis*	F	A	-	nd
15	Mt. Hope	*A*. *planirostris trinitatis*	M	J	-	nd
16	Mt. Hope	*A*. *planirostris trinitatis*	M	J	-	nd
17	Mt. Hope	*A*. *planirostris trinitatis*	M	A	100	+
18	Mt. Hope	*A*. *planirostris trinitatis*	F	A	200	+
19	Mt. Hope	*A*. *planirostris trinitatis*	M	J	-	nd
20	Mt. Hope	*A*. *planirostris trinitatis*	F	A	-	nd
21	Mt. Hope	*A*. *planirostris trinitatis*	M	J	-	nd
22	Mt. Hope	*A*. *planirostris trinitatis*	M	J	-	nd
23	Mt. Hope	*A*. *planirostris trinitatis*	F	J	-	nd
24	Mt. Hope	*A*. *planirostris trinitatis*	F	A	-	nd
25	Mt. Hope	*A*. *planirostris trinitatis*	F	J	-	nd
26	Mt. Hope	*A*. *planirostris trinitatis*	M	J	-	nd
27	Mt. Hope	*A*. *planirostris trinitatis*	M	J	-	nd
28	Lopinot	*A*. *planirostris trinitatis*	F	A	-	nd
29	Lopinot	*A*. *planirostris trinitatis*	M	J	100	-
30	Lopinot	*A*. *literatus*	F	A	200	+
31	Lopinot	*A*. *literatus*	M	J	-	nd
32	Lopinot	*A*. *literatus*	M	J	-	nd
33	Lopinot	*A*. *literatus*	F	J	-	nd
34	Lopinot	*A*. *literatus*	F	A	-	nd
35	Lopinot	*A*. *literatus*	F	A	-	nd
36	Lopinot	*A*. *literatus*	F	J	-	nd
37	Lopinot	*A*. *literatus*	F	A	400	+
38	Lopinot	*A*. *literatus*	M	J	400	+
39	Lopinot	*A*. *literatus*	F	J	-	nd
40	Lopinot	*A*. *literatus*	F	A	-	nd
41	Lopinot	*A*. *literatus*	M	A	800	+
42	Lopinot	*Glossophaga soricina*	F	A	-	nd
43	Lopinot	*Glossophaga soricina*	F	A	-	nd
44	Lopinot	*A*. *planirostris trinitatis*	M	A	200	+
45	Lopinot	*A*. *literatus*	M	A	-	nd
46	Lopinot	*A*. *literatus*	M	A	-	nd
47	Lopinot	*Sarcopteryx bilineata*	M	A	-	nd
48	Lopinot	*Glossophaga soricina*	F	A	-	nd
49	Lopinot	*Sturnira lilium*	M	A	200	+
50	Lopinot	*Sturnira lilium*	F	A	100	+
51	Lopinot	*Sturnira lilium*	F	A	-	nd
52	Lopinot	*Sarcopteryx bilineata*	M	J	-	nd
53	Lopinot	*A*. *literatus*	M	A	200	-
54	Lopinot	*A*. *planirostris trinitatis*	M	A	-	nd
55	Santa Cruz	*A*. *literatus*	M	A	-	nd
56	Santa Cruz	*A*. *literatus*	M	A	200	-
57	Santa Cruz	*Sarcopteryx bilineata*	M	A	-	-
58	Santa Cruz	*Sarcopteryx bilineata*	F	A	-	-
59	Santa Cruz	*A*. *literatus*	M	A	200	+
60	Santa Cruz	*A*. *literatus*	M	A	-	-
61	Santa Cruz	*A*. *literatus*	M	A	200	
62	Santa Cruz	*A*. *literatus*	M	A	-	nd
63	Santa Cruz	*A*. *planirostris trinitatis*	M	A	400	+
64	Santa Cruz	*A*. *literatus*	M	A	100	+
65	Santa Cruz	*A*. *literatus*	M	A	100	+
66	Santa Cruz	*A*. *planirostris trinitatis*	M	A	-	nd
67	Santa Cruz	*Sarcopteryx bilineata*	F	A	-	nd
68	Santa Cruz	*Sarcopteryx bilineata*	F	A	-	nd
69	Santa Cruz	*A*. *literatus*	M	A	200	-
70	Santa Cruz	*A*. *planirostris trinitatis*	M	A	-	+
71	Santa Cruz	*A*. *literatus*	M	A	-	-
72	Santa Cruz	*A*. *planirostris trinitatis*	M	A	400	+
73	Santa Cruz	*Sarcopteryx bilineata*	M	A	200	-
74	Santa Cruz	*A*. *literatus*	M	A	-	+
75	Santa Cruz	*A*. *literatus*	M	A	200	+
76	Santa Cruz	*A*. *literatus*	M	A	-	+
77	Maracas Valley	*C*. *perspicillata*	M	A	100	+
78	Maracas Valley	*C*. *perspicillata*	F	A	200	+
79	Maracas Valley	*A*. *planirostris trinitatis*	F	A	400	+
80	Maracas Valley	*A*. *literatus*	M	A	-	nd
81	Maracas Valley	*A*. *literatus*	M	A	-	nd
82	Maracas Valley	*C*. *perspicillata*	M	A	-	-
83	Maracas Valley	*A*. *literatus*	F	A	100	-
84	Maracas Valley	*C*. *perspicillata*	F	A	400	-
Control	673 45dpi	*A*. *jamaicensis*	+k		1600	+
Control	678 45dpi	*A*. *jamaicensis*	+k		800	+
Control	679 45dpi	*A*. *jamaicensis*	+k		1600	nd
Control	680 2dpi	*A*. *jamaicensis*	-k		-	-
Control	681 2dpi	*A*. *jamaicensis*	-k		-	nd

^1^Locations of collections are shown on Fig 2.

^2^M, male; F, female; U, undetermined

^3^Titer represents the reciprocal of the greatest dilution with signal.

^4^+, positive; -, negative; nd, not done. Insufficient sample remained to test bat 61 by western blot.

Dark grey boxes are ELISA-positive adults, whereas light grey boxes are ELISA-positive juveniles.

Cytochrome B sequencing showed that artibeus bats were *A*. *planirostris* or *A*. *literatus*; no *A*. *jamaicensis* bats were collected (data not shown). Of the six species captured, four had seropositive bats; *A*. *planirostris*, *A*. *literatus*, *Carollia perspicillata* (Seba’s fruit bat) and *Sturnira lilium* (yellow-shouldered fruit bat) (Table 2). Although *C*. *perspicillata* and *S*. *lilium* had higher seroprevalence rates than either artibeus species, the sample sizes for each was low.

**Table 2:** Seroprevalence to TCRV among adult bats of Trinidad.

Species	Diet	Ab positive (Adults)	Sampled (Adults)	Prevalence (%)
*Artibeus literatus*	frugivore	8	25	32
*A*. *planirostris trinitatis*	frugivore	8	21	38
*Carollia perspicillata*	frugivore	2	4	50
*Sarcopteryx bilineata*	insectivore	0	6	0
*Glossophaga soricina*	nectarivore	0	3	0
*Sturnira lilium*	frugivore	2	3	67

None of the captured bats had neutralizing antibodies. The pellets of lung homogenates and supernatants of inoculated Vero cells were also screened for TCRV RNA and none of the samples had amplicons (data not shown).

## Discussion

Generally speaking, suitable vertebrate reservoirs of viruses, which have been best studied in rodents [12–16], have little to no pathology and remain persistently infected for extended periods of time, perhaps for life with some viruses and their hosts. Although few studies of bats as viral reservoirs have been performed, those that have typically demonstrate this pattern [17–19]. Perhaps the best studied virus/reservoir host systems are the rodent-borne hantaviruses, which are similar to arenaviruses, in which the viruses establish apathogenic infections without eliciting aggressive immune responses [20, 21]. In each of these systems, the viruses do not cause meaningful pathology and persist for many months or longer, and heterologous hantavirus inoculation of a reservoir host species also results in innocuous infections [22, 23].

Historically, the natural reservoir host(s) of TCRV was presumed to be artibeus bats [1, 3]. All other mammarenaviruses with known reservoirs are hosted by rodents [24], thus the hypothesis that bats may serve as a reservoir of TCRV is peculiar. We have obtained serological evidence that TCRV or similar arenavirus is circulating in at least two, and possibly four species of fruit bats in Trinidad; flat-faced fruit bats, great fruit-eating bats, Seba’s fruit bats and yellow-shouldered fruit bats. Flat-faced fruit bats, great fruit-eating bats and yellow-shouldered fruit bats have previously been identified as having antibodies to TCRV [1, 3] but detection of antibodies in Seba’s fruit bat has not been reported until now. We did not capture any Jamaican fruit bats (*A*. *jamaicensis*), which is one of the two species originally identified as a host when TCRV isolates were first made in the 1950s [1]. Considering other evidence that Jamaican fruit bats are not found in Trinidad [4], it is likely that flat-faced fruit bats were misidentified as Jamaican fruit bats in the original paper.

Viral RNA was not detected in the lung tissues of bats or in Vero cells inoculated with clarified lung homogenates, nor was virus isolated from blind passage on Vero cells, suggesting none of the bats were infected at the time of capture. We previously performed experimental infections of Jamaican fruit bats with TCRV TRVL-11573 and determined that high doses of virus caused disease with high mortality rates but that low dose virus resulted in clearance without conspicuous disease [7]. Interestingly, surviving bats in this study had only modest antibody titers by ELISA (Table 1) and very low neutralizing titers [7]. Little brown bats (*Myotis lucifugus*) do not appear to substantially use somatic hypermutation (SHM), suggesting that affinity maturation (which accounts for high antibody titers) may be limited [25]. If SHM is limited in artibeus and other bat species, it could account for the low titers we observed in this study.

Considering the serological evidence presented here that at least four species of fruit bats, and a previous study showing other bat species [3], are susceptible to arenaviruses, it may be that bats are spillover hosts from rodent reservoirs. The high seroprevalence suggests that after spillover the virus may be transmitted among bats and considering the high densities and direct contact among individuals within a colony, transmission could occur. However, experimental infections previously performed by us showed that despite detectable vRNA in oral and rectal swabs for several weeks, transmission to sentinel bats did not occur [7]. It is possible that the artificial housing in our experimental model disrupted the natural biology of the bats (e.g., confined to cages, behavioral changes, dietary differences, etc.) that may have prevented transmission.

Sixty-eight species of bats are found in Trinidad and Tobago [26], which are about 27 km apart, thus, we cannot exclude the possibility that another bat species in Trinidad may be a reservoir host of TCRV. It is also possible the bats were infected with another arenavirus that could not be detected with the TCRV-specific primers we used; however, we did not observe cytopathic effect in the Vero cells, which are routinely used for arenavirus propagation. Trinidad is 12 km from Venezuela and it is possible that TCRV may move between Trinidad and Venezuela [27], where rodent-borne arenaviruses are found, including Gunarito virus and Pirital virus [24, 28, 29]. The next closest nation to Trinidad and Tobago is Grenada, where artibeus bats are also found. However, the distance between the islands is about 145 km, making it difficult for artibeus bats to routinely migrate between the islands because their range is about 15 km [27].

The recent isolation of TCRV from lone star ticks in central Florida [8] also suggests that artibeus bats are not reservoir hosts. Lone star ticks are not known to feed on bats; however, they routinely feed on rodents and other terrestrial mammals [30]. Other species of amblyoma ticks are found in Trinidad but nothing is known about the viruses they may harbor [31]. Trinidad and mainland Florida do not share common species of rodents or bats, other than introduced house mouse (*Mus musculus*) and Norway rats (*Rattus norvegicus*), neither of which have been shown to host TCRV. Several Cricetidae rodent species are found in Trinidad and Florida, and many arenaviruses are hosted by members of this family. Insectivorous velvety free-tailed bats (*Molossus molossus*) are common in Trinidad but found only in the Florida Keys, suggesting it is not the source of TCRV isolated from central Florida. TCRV may have multiple natural reservoir host species, which is unusual but not unprecedented among the mammarenaviruses [24]. The original paper describing TCRV’s isolation did not identify rodents as potential reservoirs; however, it is unclear as to how many species and individuals were sampled [1]. Thus, the accumulated evidence to date suggests that artibeus bats are not reservoir hosts of Tacaribe virus.

## Conclusions

Artibeus bats were thought to be reservoir hosts of Tacaribe virus because they had been only isolated from bats of this genus. We collected bats in Trinidad and found serological evidence of arenavirus circulation among frugivorous, but not insectivorous, bats. No virus was isolated nor viral RNA detected in the bats, suggesting infection leads to clearance in bats that survive. These results add to the accumulating evidence that suggests artibeus bats are not reservoir hosts of Tacaribe virus.
